# The diversity of citrus endophytic bacteria and their interactions with *Xylella fastidiosa* and host plants

**DOI:** 10.1590/1678-4685-GMB-2016-0056

**Published:** 2016-10-10

**Authors:** João Lúcio Azevedo, Welington Luiz Araújo, Paulo Teixeira Lacava

**Affiliations:** 1 Departamento de Genética, Escola Superior de Agricultura Luiz de Queiroz, Universidade de São Paulo, Piracicaba, SP, Brazil.; 2 Departamento de Microbiologia, Instituto de Ciências Biomédicas, Universidade de São Paulo, São Paulo, SP, Brazil.; 3 Departamento de Morfologia e Patologia, Centro de Ciências Biológicas e da Saúde, Universidade Federal de São Carlos, São Carlos, SP, Brazil.

**Keywords:** endophytes, Citrus sinensis, Curtobacterium flaccumfaciens, Methylobacterium mesophilicum, symbiotic control

## Abstract

The bacterium *Xylella fastidiosa* is the causal agent of citrus variegated chlorosis (CVC) and has been associated with important losses in commercial orchards of all sweet orange [*Citrus sinensis* (L.)] cultivars. The development of this disease depends on the environmental conditions, including the endophytic microbial community associated with the host plant. Previous studies have shown that *X. fastidiosa* interacts with the endophytic community in xylem vessels as well as in the insect vector, resulting in a lower bacterial population and reduced CVC symptoms. The citrus endophytic bacterium *Methylobacterium mesophilicum* can trigger *X. fastidiosa* response *in vitro*, which results in reduced growth and induction of genes associated with energy production, stress, transport, and motility, indicating that *X. fastidiosa* has an adaptive response to *M. mesophilicum*. Although this response may result in reduced CVC symptoms, the colonization rate of the endophytic bacteria should be considered in studies that intend to use this endophyte to suppress CVC disease. Symbiotic control is a new strategy that uses symbiotic endophytes as biological control agents to antagonize or displace pathogens. Candidate endophytes for symbiotic control of CVC must occupy the xylem of host plants and attach to the precibarium of sharpshooter insects to access the pathogen. In the present review, we focus on interactions between endophytic bacteria from sweet orange plants and *X. fastidiosa*, especially those that may be candidates for control of CVC.

## Endophytic microorganisms and biological control

Endophytes can be isolated from surface-disinfected plant parts or the inner plant tissues and are defined as microorganisms, mainly bacteria and fungi, that live within a plant for at least a part of their life cycle without causing apparent harm to the host (Petrini *et al*., 1989; Hallmann *et al*., 1997; Azevedo *et al*., 2000). A more comprehensive definition was proposed by Azevedo and Araújo (2007), who described endophytes as all microorganisms that may or may not be successfully cultured, internally colonize the host plant and do not cause apparent damage and/or visible external structures. This definition was amended by Mendes and Azevedo (2008) to divide endophytes in two types: type I, which does not produce external structures, and type II, which produces external structures, such as nodules from nitrogen-fixing bacteria and fungi-plant mycorrhizal associations. Recently, Hardoin *et al*. (2015) proposed that the term “endophyte” should be used as a habitat only, not a function, including all microorganisms able to colonize the inner plant tissues.

Endophytes have been reported to contribute to host plant protection and, ultimately, survival (Sturz and Matheson, 1996; Hallmann *et al*., 1998; Azevedo *et al*., 2000; Newman and Reynolds, 2005; Rosenblueth and Martinez-Romero, 2006; Reinhold-Hurek and Hurek, 2011; Suryanarayanan, 2013; Nair and Padmavathy, 2014; Podolick *et al*., 2015). Because endophytes colonize an ecological niche similar to that of phytopathogens, they are possible biocontrol agents (Hallmann *et al*., 1997; Hardoin *et al*., 2015). The potential for practical applications of endophytes has led to studies investigating the ability of bacteria to control both disease and insect infestations, as well as promote plant growth (Azevedo *et al*., 2000; Kozdrój *et al*.; 2004; Kavino *et al*., 2007; Podolick *et al*., 2015). Indeed, previous work has suggested that endophytic microorganisms have the potential to control pathogens (Sturz and Matheson, 1996; Duijff *et al*., 1997; Sharma and Nowak, 1998; Sturz *et al*., 1998; Lacava *et al*., 2004; 2007a), insects (Petrini *et al*., 1989; Azevedo *et al*., 2000), and nematodes (Hallmann *et al*., 1997). In grass, infection by endophytic fungi and alkaloid production reduced aphid feeding but had no effect on viral titers in the host plant (Ruá *et al*., 2013). However, the authors observed that the virulence of the viral infection was reduced in endophyte-infected plants, suggesting that although the endophyte had no effect on viral infection, the presence of the endophytes could trigger a host response that reduced virulence. The balance of this interaction among the host plant, endophytic fungi, aphids and viruses was influenced by the host and endophyte genotypes and was also driven by environmental conditions (Ruá *et al*., 2013; 2014).

In addition, endophytes can also accelerate seed emergence, assist in establishing the plant under adverse conditions (Chanway, 1997), and increase plant growth and development (Bent and Chanway, 1998; Lazarovits and Nowak, 1997; Pillay and Nowak, 1997; Nassar *et al*., 2005, Bao and Roossinck, 2011; Bezerra *et al*., 2013; Verma *et al*., 2015; Chebotar *et al*., 2015).

Endophytic bacteria may play a significant role in protection against plant pathogens and in the overall productivity of an agricultural ecosystem (Hallmann *et al*., 1997; Sturz *et al*., 2000). These microorganisms produce molecules that function as growth-promoting metabolites, insect-pest repellents, antimicrobials against plant pathogens, and protectants against stress (Rai *et al*., 2014). They can also produce unique secondary metabolites that can be exploited in pharmaceutical, agricultural and other industries (Golinska *et al*., 2015).

The utilization of endophytic bacteria for biotechnological purposes has recently increased, especially with regard to insect and disease control and plant growth promotion. Endophytic bacteria promote plant growth in three major ways, by synthesizing compounds that are useful to the plants, by facilitating the uptake of certain nutrients from the soil, and by controlling or preventing disease (biological control). Growth promotion mediated by endophytic bacteria occurs via several mechanisms: the synthesis of enzymes; the production of hormones such as auxin [indoleacetic acid (IAA)]; symbiotic nitrogen fixation; antagonism against phytopathogens by siderophores, chitinases or antibiotics; and the solubilization and mineralization of nutrients, particularly insoluble mineral phosphates (Lacava and Azevedo, 2013, 2014). Indeed, interactions between endophytes and plants can promote plant health and play a significant role in low-input sustainable agriculture for both food and nonfood crops. Nonetheless, an understanding of the mechanisms that enable endophytes to interact with plants is essential for realizing the biotechnological potential of these microorganisms (Quecine *et al*., 2014).

In citrus trees, the endophytic environment becomes more stable and uniform over time. This may result from selection of particular genotypes within each local microbial population (Araújo *et al*., 2002). Consequently, bacteria living in an endophytic environment may adapt to this more stable environment, resulting in intense interactions among the populations (Lacava *et al*., 2004).

Several reports highlighted the relationships among bacterial populations and suggest that CVC symptoms in citrus plants could be influenced by the population balance among *Methylobacterium* spp., *Curtobacterium flaccumfaciens* and *X. fastidiosa* (Araújo *et al*., 2002; Lacava *et al*., 2004, 2006b, 2007a; Ferreira Filho *et al*., 2012). Understanding the relationship among endophytic bacteria within sweet orange trees and *X. fastidiosa* may lead to strategies to control CVC using endophytic bacteria (Ferreira Filho *et al*., 2012) or inducing physiological shifts in both the host plant and microbial community that result in more balanced and stable interactions.

## The plant pathogen *Xylella fastidiosa*

The first report of symptoms caused by what we now call *Xylella fastidiosa* was in 1884 in the grape-growing region of southern California (US). A disease syndrome, known today as Pierce's disease (PD), was later described in detail (Pierce, 1892). Subsequently, similar diseases were reported in many fruit tree and ornamental species, especially in North and South America (Hopkins, 1989). *X. fastidiosa* is a fastidious, Gram-negative, xylem-limited, rod-shaped bacterium with distinctive rippled cell walls. It is non-flagellated, does not form spores and measures 0.1-0.5 1-5 μm (Nyland *et al*., 1973; Bradbury, 1991). This Gram-negative bacterium was formally named only in 1987 (Wells *et al*., 1987). It is extremely slow-growing in culture. These traits have made the pathogen difficult to study and contributed to its previous obscurity. The taxonomic position of *X. fastidiosa* (Wells *et al*., 1987) is: Bacteria, *Gracilicutes*, aerobic rods, Category I, Group 4, Subgroup 4 A (Holt, 1994). Natural transmission occurs via insects feeding suctorially on xylem sap. Transmission efficiency varies widely among vector species. The bacterium overwinters in the xylem of the host plants as well as in weeds (Lopes *et al*., 2003; Wistrom and Purcell, 2005).

*X. fastidiosa* (Wells *et al*., 1987) resides in the xylem vessels of a broad range of perennial plants in the New World and has been shown to cause important diseases in a variety of fruit trees and vines. These include PD in grapevines (Davis *et al*., 1981; Hopkins and Purcell, 2002), leaf scorches in pecan (Sanderlin and Heyderich-Alger, 2000; Sanderlin and Melanson, 2006), pear (Leu and Su, 1993), plum (Raju *et al*., 1983), almond (Mircetich *et al*., 1976), mulberry (Kostka *et al*., 1986), elm, sycamore, oak (Hearon *et al*., 1980), maple (Sherald *et al*., 1987), coffee (de Lima *et al*., 1998), oleander and olives (Saponari *et al*., 2013), as well as alfalfa dwarf (Goheen *et al*., 1979), phony peach disease (Wells *et al*., 1981), periwinkle wilt (McCoy *et al*., 1978), and citrus variegated chlorosis (Chang *et al*., 1993; Hartung *et al*., 1994). Strains of *X. fastidiosa* have a wide host range in the native flora, where they exist without inducing symptoms of disease, and they are transmitted by common sharpshooter insects (Freitag, 1951; Freitag and Frazier, 1954).

Following the publication of the *X. fastidiosa* genome (Simpson *et al*., 2000), there was an increased number of articles describing new features of *X. fastidiosa*, such as the existence of plasmids and phages and the discovery of conjugation and recombination in the species as well similarities among genomes of distinct *X. fastidiosa* strains. Bhattacharyya *et al*. (2002) described a whole-genome comparative analysis of three phytopathogenic strains from almond, citrus and oleander. This study demonstrated that these genomes are closely related. Van Sluys *et al*. (2003) described limited genomic structural variability within *X. fastidiosa*, which suggested that phylogenetic groups colonizing different host plants have similar pathogenicity mechanisms. Varani *et al*. (2012) showed that comparative genomic databases were an important information resource to explore the annotation, genomic features and biology of different *X. fastidiosa* strains. Other results published after the *X. fastidiosa* genome release should also be mentioned. Marques *et al*. (2001) described the sequence of the plasmid pXF51 from the plant pathogen *X. fastidiosa*, showing that this plasmid contained genes for conjugation, replication and mobilization but apparently had no role in pathogenesis, only in conjugative transfer. Nunes *et al*. (2003) constructed a microarray and evaluated the occurrence of prophages, plasmids and genomic islands (18% of the genome). The authors showed that most of these elements are transcriptionally active and could explain the ability of *X. fastidiosa* strains to infect a wide range of plant species. Plasmids were also found and sequenced by other authors, such as Rogers and Stenger (2012). Additionally, Kung and Almeida (2011) demonstrated that recombination can occur at relatively high rates and may play a large role in shaping the genetic diversity of *X. fastidiosa*. Chen and Civerali (2008) demonstrated that phages particles are released by *X. fastidiosa* cultures for the first time, and Summer *et al*. (2010) carried out genomic and bacterial analyses of the phage Xfas53 and related prophages of *X. fastidiosa*. Guilhabert and Kirkpatrick (2003) successfully transformed *X. fastidiosa* using a broad host range plasmid.

However, despite the increasing knowledge of *X. fastidiosa* characteristics, the molecular mechanisms determining host plant specificity have not been elucidated (Almeida and Nunney, 2015). The interactions between *X. fastidiosa* and attacked plants were discussed in more detail in a review by Hopkins and Purcell (2002). More recently, Almeida and Nunney (2015) reviewed the main processes that led to the emergence of the diseases caused by *X. fastidiosa*. Since 2002, it has become clear that the frequency and density of the pathogens and endophytic bacteria in citrus plants may be a result of a tripartite interaction associated with environmental conditions (Araújo *et al*., 2001, 2002). Therefore, in the present review, the CVC status and the molecular and ecological aspects of this tripartite interaction will be discussed. In addition, a possible strategy based on symbiotic control will be proposed.

## Citrus variegated chlorosis (CVC)

Citrus variegated chlorosis (CVC) is a disease of the sweet orange [*Citrus sinensis* (L.)], which is caused by *X. fastidiosa* (Chang *et al*., 1993; Hartung *et al*., 1994; Schaad *et al*., 2004), a phytopathogenic bacterium that has been shown to infect all sweet orange cultivars (Li *et al*., 1997a). CVC was first reported in Brazil in 1987 and has rapidly become one of the economically most important diseases affecting sweet orange production in Brazil (Rossetti *et al*., 1990; Lee *et al*., 1991). CVC rapidly spread to most major citrus-growing areas through unregulated movement of infected nursery stock due to a previous lack of certification programs and high CVC infection rates in Brazil.

Brazil is the largest producer of citrus fruits in the world (25% of the total world production), supplying most of the international market with concentrated orange juice. More than 80% of these products are produced in the state of São Paulo. Approximately 15 years ago, CVC was found in at least 90% of the orchards in Brazil (Lambais *et al*., 2000). The incidence and severity of CVC are higher in the northern region than in the southern region of São Paulo (Ayres *et al*., 2001; Gonçalves *et al*., 2014). In 2011, more than 40% of the sampled plants in Brazil had CVC symptoms, approximately 5% more than in 2010, showing that the disease is still increasing (www.agriculture.gov.br/arq_editor/file/camaras_exterior).

CVC is considered one of the most important diseases affecting the Brazilian citrus industry, and economic losses due to CVC can reach $120 million per year (Bové and Ayres, 2007). In an effort to reduce losses, additional regulations have been placed on the production and commercialization of citrus seedlings. In 2003, it became mandatory in São Paulo to propagate citruses in protected, screened houses, increasing the cost of production (Carvalho, 2003). CVC affects mostly oranges (*C. sinensis*); it has mainly been observed on the cultivars ‘Pera’, ‘Hamlin’, ‘Natal’ and ‘Valencia’. It occurs in trees propagated on all commonly used rootstocks in Brazil: *C. limonia*, *C. reshni* and *C. volkameriana* (Li *et al*., 1997c). The disease has not been observed in limes (*C. latifolia*) or mandarins (*C. reticulata*), even when the trees were planted in severely affected orange groves (Li *et al*., 1997b). Some weed species are also hosts and act as reservoirs of infection (Smith *et al*., 1997). This disease continues to show an increase in severity, as mentioned, with 35%-40% of the sweet orange trees in São Paulo, Brazil currently showing yield losses (www.fundecitrus.com.br). Citrus plants with CVC show a notable leaf chlorosis, similar to zinc deficiency, as the initial symptom (Laranjeira *et al*., 1998; Machado *et al*., 2006). Later symptoms include wilting, canopy dieback, necrotic leaf lesions, and undersized, hard fruit (Derrick and Timmer, 2000; Hopkins and Purcell, 2002). The causal agent of CVC has been found to be transmitted by sharpshooter leafhoppers (Cicadellidae) in Brazil (Lopes *et al*., 1996; Almeida and Purcell, 2003). CVC has been experimentally transmitted by 11 different sharpshooter species tested in Brazil (www.fundecitrus.com.br). Additionally, the pathogen can be transmitted through seeds (Li *et al*., 2003). Although *X. fastidiosa* was the first plant pathogen to have its genome sequenced (Simpson *et al*., 2000), there is still no effective control for CVC. The pathogen is known to have an extraordinary host range among higher plants in New World ecosystems (Freitag, 1951). Interestingly, within the majority of native host plants, *X. fastidiosa* does not damage the host plant and behaves as an endophyte (Purcell and Saunders, 1999). In contrast, the horticultural crops that suffer from diseases caused by *X. fastidiosa* are those that have been introduced into New World ecosystems (Chen *et al*., 2000). The observation that a few asymptomatic trees persist in some infected orchards may lead to new approaches to the control of CVC. These asymptomatic plants have the same genotype as diseased plants and are located in the same grove under similar climatic and edaphic conditions, suggesting that some other factor is responsible for resistance to CVC. One factor that may influence the resistance to CVC is the nature of the endophytic microbial community colonizing individual *C. sinensis* plants (Araújo *et al*., 2002).

## Endophytic bacteria from citrus plants and interactions with *Xylella fastidiosa*

We have focused on the interaction between members of the endophytic bacterial community, such as *Methylobacterium* spp. and *C. flaccumfaciens*, which occupy the same ecological niche as *X. fastidiosa* in the xylem vessels of citrus plants (Araújo *et al*., 2002). The genus *Methylobacterium* is classified in the α2 subgroup of the Proteobacteria and includes a group of strictly aerobic, Gram-negative, pink-pigmented, facultatively methylotrophic (PPFM) bacteria characterized by their ability to utilize single-carbon compounds, such as methanol and formaldehyde, via the serine pathway, as well as a wide range of multi-carbon growth substrates (Green, 1992; Urakami *et al*., 1993; Wood *et al*., 1998; Doronina *et al*., 2000, 2002; McDonald *et al*., 2001; Van Aken *et al*., 2004; Anesti *et al*., 2004; Van Jourand *et al*., 2004; Gallego *et al*., 2005a,b,c, 2006). In the host plant, during plant interactions, biofilm formation on surface of the root and hypocotyl of *C. roseus* occurred prior to endophytic colonization (Andreote *et al*., 2006). In addition, the authors observed that *M. mesophilicum* SR1.6/6 induced shifts in the indigenous endophytic α- and β-Proteobacteria populations, using DGGE techniques. In soybean, Araújo *et al*. (2015) observed that during the initial step of plant colonization (including plant exudate recognition and adaptation), based on transcriptomic analysis, several genes involved in membrane transport were expressed, suggesting metabolic activation in the presence of root exudate. In addition, the results showed that genes encoding proteins related to suppression of oxidative stress, such as glutathione peroxidase and glutathione synthetase, were induced, suggesting that these genes are probably related to cellular detoxification during plant root colonization. Additionally, Dourado *et al*. (2013) showed that bacterial density is an important characteristic during plant colonization because some genes related to metabolism, stress and pathogenesis were induced by quorum-sensing systems, indicating that plant colonization depends on bacterial coordination of events related to host recognition and stress suppression.

Araújo *et al*. (2001) isolated several endophytic bacteria from different citrus rootstocks and showed that *Alcaligenes* sp., *Bacillus* spp. (including *B. cereus*, *B. lentus*, *B. megaterium, B. pumilus*, and *B. subtilis*), *Burkholderia cepacia, Curtobacterium flaccumfaciens, Enterobacter cloacae, Methylobacterium extorquens*, and *Pantoea agglomerans* were the dominant species. Furthermore, the frequency of endophytic bacteria in healthy, escape, and CVC-affected *Citrus sinensis* plants was studied using cultivation as well as cultivation-independent methods (Araújo *et al*., 2002). Bacteria from the genus *Methylobacterium* were the most frequently isolated endophytes from CVC-symptomatic citrus plants (*C. sinensis*) (Araújo *et al*., 2002; Lacava *et al*., 2004, 2006a,b); however, Araújo *et al*. (2002) observed that *M. extorquens* was only isolated from CVC-affected plants while *M. mesophilicum* was isolated from healthy plants, indicating that specific *Methylobacterium* species may be related to the citrus plant physiological condition. Furthermore, in *in vitro* studies, Lacava *et al*. (2004) showed that *M. mesophilicum* could reduce the growth of *X. fastidiosa*, while *M. extorquens* had no effect on the *X. fastidiosa* growth. In addition, in co-inoculation experiments using *Catharanthus roseus* as a model plant, Lacava *et al*. (2006a) showed that the population of *M. mesophilicum* was lower in the presence of *X. fastidiosa* compared to inoculation of this endophytic bacterium alone and that the population of *X. fastidiosa* was in turn reduced by the inoculation of *M. mesophilicum*. The results suggest that these bacteria could compete for nutrients and colonization sites inside the host plant. In fact, using microarray analysis, Dourado *et al*. (2015) showed that the *M. mesophilicum* strain SR1.6/6 directly down-regulated genes related to bacterial growth, such as DNA replication and protein synthesis genes (50S ribosome protein and topoisomerase enzyme genes) in *X. fastidiosa*. In contrast, *C. flaccumfaciens* strain ER1/6, another citrus endophyte, up-regulated genes related to protein synthesis. Additionally, despite the *X. fastidiosa* growth reduction, genes related to energy generation (fumarate hydratase and dihydrolipoamide dehydrogenase from the Krebs cycle) were up-regulated in *X. fastidiosa* in response to *M. mesophilicum*, suggesting that although the CVC causal agent is not growing, energy is necessary to maintain the interaction profile, including genes related to stress response and membrane transporters.

Therefore, the development of CVC symptoms in citrus plants could be influenced by the population balance among the endophytic bacteria *Methylobacterium* spp. and *X. fastidiosa* (Lacava *et al*., 2004; Dourado *et al*., 2015) and environmental conditions, which affect the host physiology and response to the presence of the microbial community.

The genus *Curtobacterium* has been defined by Yamada and Komagata (1972) as Gram-positive aerobic bacteria, with some so-called motile brevibacteria. *Curtobacterium* strains have been isolated from rice and other plants, and *C. flaccumfaciens*, in particular, is a well-established plant pathogen (Collins and Jones, 1983). However, *Curtobacterium* has been isolated as an endophyte from many crops, including red clover (Sturz *et al*., 1998), potato (Sturz and Matheson, 1996), yam (Tor *et al*., 1992), prairie plants (Zinnier *et al*., 2002), and citrus (Araújo *et al*., 2001). Several reports have indicated that *C. flaccumfaciens* can function as a biological control agent against many pathogens and may function either by triggering induced systemic resistance (Raupach and Kloepper, 1998) or by antibiosis (Sturz and Matheson, 1996).

The bacterium *C. flaccumfaciens* has been more frequently isolated from CVC-asymptomatic than from CVC-symptomatic orange (*C. sinensis*) and tangerine (*Citrus reticulata*) plants (Araújo *et al*., 2002; Lacava *et al*., 2004), and it was also suggested, based on *in vitro* interaction experiments, that the growth of *X. fastidiosa* could be inhibited by endophytic *C. flaccumfaciens*. Symptoms of *X. fastidiosa* infection in *C. roseus*, such as shortened internodes, reduced flowering and stunting and chlorosis of leaves with occasional scorch symptoms, were reduced or prevented entirely by co-inoculation with *C. flaccumfaciens* (Lacava *et al*., 2007a). Madagascar periwinkle, *C. roseus* (L.) G. Don, has been identified as an excellent experimental host for *X. fastidiosa* (Monteiro *et al*., 2001). Symptoms of *X. fastidiosa* infection in periwinkle include shortened internodes, reduced flowering, stunting, and leaf chlorosis with occasional scorch symptoms and wilting (Monteiro *et al*., 2001). In comparison with the sweet orange, the Madagascar periwinkle is significantly easier to maintain in a greenhouse, and symptom induction following inoculation with *X. fastidiosa* is both more rapid and more reliable. The Madagascar periwinkle has also been utilized to study the interactions between *X. fastidiosa* and other endophytic bacteria (Lacava *et al*., 2006a, 2007a).

## Biological control of CVC

Lacava *et al*. (2004) reported that the growth of *X. fastidiosa* was inhibited by endophytic *C. flaccumfaciens* *in vitro*, and Lacava *et al*. (2007a) demonstrated that *C. flaccumfaciens* reduced the severity of disease symptoms when co-inoculated with *X. fastidiosa* in periwinkle (*C. roseus*) plants.

Isolation and denaturing gradient gel electrophoresis (DGGE) techniques revealed several genera of bacteria as colonizers of glassy-winged sharpshooter (GWSS) heads. These bacteria were identified by 16S sequencing and included *M. extorquens* and *C. flaccumfaciens*. The GWSS *Homalodisca vitripennis* Germar (Hemiptera: Cicadellidae) [formerly *H. coagulata* (Takiya *et al*., 2006)] is the most widespread sharpshooter insect vector of *X. fastidiosa* in the United States. In addition, Kirkpatrick and Wilhelm (2007) have also isolated strains of *C. flaccumfaciens* as part of the endophytic bacterial community of grapevines in California. In Brazil, *C. flaccumfaciens* is consistently isolated as an endophytic bacterium from citrus plants (Araújo *et al*., 2002; Lacava *et al*., 2004).

It is likely that endophytic bacteria are introduced into sweet orange trees by sharpshooter insects in the same manner as *X. fastidiosa*. Gai *et al*. (2011) showed that *Curtobacterium* sp. were the most important bacteria colonizing the heads of the insect vectors of *X. fastidiosa* in Brazil.

*Curtobacterium flaccumfaciens* was shown to play an important role in the prevention of CVC symptoms in citrus trees (Araújo *et al*., 2002; Lacava *et al*., 2004, 2007a). The presence of the citrus endophyte *Curtobacterium* sp. in insect heads could explain why the transmission efficiency of *X. fastidiosa* by vectors is low (5 to 10%) compared to the transmission of *X. fastidiosa* subsp. *fastidiosa* by GWSS, which transmit PD (45%) (Krügner *et al*., 2000; Redak *et al*., 2004).

Endophytic bacteria could influence disease development by reducing the efficiency of transmission by insects due to competition with pathogens in host plants and also in insect foreguts (Gai *et al*., 2011). In addition, the bacterial communities in the foregut of insect vectors of *X. fastidiosa* change with time and environmental conditions and in different insect species. However, because members of the genus *Curtobacterium* were consistently detected in the insect vectors of *X. fastidiosa* (Gai *et al*., 2011), they may be candidates for biological control of *X. fastidiosa*, which requires endophytic bacteria (Lacava *et al*., 2007a) that can colonize both the insect vectors of CVC and citrus plants.

## Symbiotic control (SC)

The technique of paratransgenesis was developed as a novel method to create conditions that render insect vectors incompetent. The symbiotic control (SC) strategy employs both paratransgenic (defined below) and non-recombinant methods to control disease or health problems. In some cases, these solutions may result in competitive displacement of the pathogen with a more benign microbe.

Paratransgenesis was developed to prevent the transmission of pathogens from insect vectors to humans (Beard *et al*., 1998, 2001, 2002; Rio *et al*., 2004; Hurwitz *et al*., 2011). The key concept in paratransgenesis is the genetic alteration of symbiotic microbes that are carried by insects (therefore, they are paratransgenic insects). The genetic alterations of the symbiotic microbes are designed to increase their competitiveness in the insect vector at the expense of the pathogen. This overall strategy of disease prevention is an example of SC and is a variation on the theme of symbiotic therapy (Ahmed, 2003; Hurwitz *et al*., 2011). Genetic manipulation has fitness costs that must be factored in to the application (Durvasula *et al*., 1997; Miller, 2007, 2011).

The key to SC, and therefore paratransgenesis, is to find a local candidate microbe with an existing association with the pathosystem that is being investigated. The local candidate microbe should occupy the same niche as, or have access to, the target pathogen or condition (Durvasula *et al*., 1997; Hurwitz *et al*., 2011). The local origin of the biocontrol microbe in SC differs from classical biological control, where microbes, herbivores, parasites or predators are sought from outside the local ecosystem for establishment in the local ecosystem to control a pest, such as a plant or invertebrate (Miller, 2011; Hurwitz *et al*., 2011). In SC, all elements originate at the local site and have already co-evolved with and been established in the pathosystem; foreign exploration is not only unnecessary but also most likely counter-productive. Because of these strict requirements, a suitable symbiotic candidate may not always be found or may not be amenable to practical manipulation (Miller, 2011; Hurwitz *et al*., 2011).

Microbes chosen for symbiotic control must be able to pass subsequent regulatory scrutiny (Miller, 2011; Bourtzis *et al*., 2012). Once a candidate symbiont is identified as a control agent for paratransgenesis, all genetic or other manipulations can be local. Indeed, a symbiotic control or paratransgenic solution developed for a specific location may not be suitable for another site or condition elsewhere (Durvasula *et al*., 1999, 2003; Miller, 2011).

Once a microbe is identified as having potential for symbiotic or paratransgenic control, it is studied to define the requirements for culture and reintroduction into the pathosystem and the suitability for genetic alteration, if necessary. The methods selected must be adaptable to ordinary practices in the target area. In the case of paratransgenic control, a gene or genes to be introduced into the endosymbiont to influence its interaction with the pathogen must be identified. Beard *et al*. (2001) isolated and characterized symbiont bacteria from various triatomine species, which are vectors of Chagas disease, and developed a method for genetically transforming them. These authors reintroduced them into triatomine species, thereby producing stable paratransgenic insects that express heterologous gene products.

Pierce's disease (PD) was first detected in Southern California in 1884, where it destroyed approximately 40,000 acres of grapes in Anaheim, CA during a 5-year outbreak (Pierce, 1892; Goodwin and Purcell, 1992). After this devastating experience, PD became only an occasional concern to West Coast viticulture for decades until the mid-1990s, when the GWSS became established in California. The GWSS is a major concern for horticultural industries beyond viticulture due to its ability to transmit *X. fastidiosa* strains causing scorch diseases in a number of host plants, including *X*. *fastidiosa* subsp. *fastidiosa* that causes PD in grapevines (Wistron and Purcell, 2005). As with other sharpshooter insects, *H. vitripennis* is a xylophagous insect that feeds on hundreds of plant species (Purcell and Hopkins 1996; Purcell and Saunders 1999); citrus is one of its preferred hosts (Blua *et al*., 2001). Perring *et al*. (2001) demonstrated a relationship between PD incidence in grapes and the proximity of vineyards to citrus orchards. This leafhopper, which can serve as a vector of *X. fastidiosa*, has the capacity to feed on more than 70 different plant species and can survive winter temperatures as low as −6 °C (Park *et al*., 2006). Moreover, compared with other *X. fastidiosa*-carrying insects associated with PD that are native to California, GWSS has a longer flight range (up to a quarter mile). These traits make the GWSS a very serious threat to the wine industry of southern and central California (Castle *et al*., 2005). Indeed, since the first identification of GWSS in California vineyards, programs aimed at controlling the dissemination of this insect to prevent PD outbreaks have involved more than US$ 160 million of direct investments (http://www.cdfa.ca.gov/phpps/pdcp/). Control of any of the GWSS-transmitted diseases of horticultural crops in California by an SC or paratransgenic approach would be of immediate interest to other industries as well. The objective or rationale for developing a method of SC for PD is to disrupt vector transmission with minimal effects on other crops. SC would be available to local vineyards for local control instead of the area-wide treatments of alternative host plants that are currently used. Treatment of citrus with systemic insecticides for GWSS to reduce the chance of acquiring and spreading pathogens in adjacent vineyards cannot be considered a long-term solution. SC would be more selective and have fewer side effects than other biological control practices. The SC organisms inhabit the xylem fluid of the target plants yet do not contaminate the berries of the grapevines. It remains to be determined if one treatment would be effective for an entire season (Miller, 2011).

Three potential bacterial candidates, *Alcaligenes* sp., *Chryseomonas* sp., and *Ralstonia* sp., for SC of PD were collected from GWSS in southern California (Bextine *et al*., 2004). All were endophytes transmitted to different host plants by GWSS in a manner analogous to the pathogen; thus, the candidates had access to the pathogen in host plants or in the insect vector, providing the needed access. *Alcaligenes denitrificans* var. *xylosoxidans* (Axd) was selected for further development because the endophytic bacterium should have most of the requirements for a successful paratransgenesis strategy such as: a) a population of microbes that is amenable to culture and genetic manipulation *in vitro* must exist within a disease-transmitting vector; b) facile methods for isolating and transforming the endophytic bacteria must be present; c) transformation of the symbiotic/endophytic bacteria must result in stable mutants; d) genetic manipulation of the bacteria should not affect their symbiotic functions in the host vector; e) genetic manipulation of symbiotic bacteria should not render them virulent, either to the target vector or other organisms in the environment. Furthermore, bacteria chosen as gene-delivery vehicles must not be pathogens themselves.

Successful delivery to and colonization of Axd in the foregut regions of GWSS suggest that a paratransgenic approach to manage, prevent, and/or control Pierce's disease is possible (Bextine *et al*., 2004).

Lacava *et al*. (2007b) used isolation and denaturing gradient gel electrophoresis (DGGE) techniques to identify several genera of bacteria as colonizers of the heads of GWSS collected in orange groves. As identified by 16S rRNA sequencing, these included *Bacillus*, *Cryocola*, *Microbacterium*, *Micrococcus* and *Pedobacter*. In addition, *Methylobacterium extorquens*, *Curtobacterium flaccumfaciens*, *Baumannia cicadellinicola* and various *Pseudomonas* and *Wolbachia* species were found. Of these genera, *Bacillus*, *Pseudomonas*, *Methylobacterium* and *Curtobacterium* were previously described as endophytes that are able to colonize citrus plants. The work of Araújo *et al*. (2002) strongly suggested that there are interactions among *Methylobacterium* spp., *C. flaccumfaciens* and *X. fastidiosa*. These results reinforced the idea that all of these bacteria could interact in the insect vector as well as in the host plant.

Furthermore, Lacava *et al*. (2004) suggested that CVC symptoms in citrus plants could be influenced by the interactions among these three species. In a study of the diversity of bacterial communities associated with GWSS foreguts, they used culture-dependent methods as well as procedures based on sequence polymorphisms (DGGE) of the 16S rRNA gene in total DNA extracted from GWSS foreguts. Lacava *et al*. (2007b) suggested that the diversity profiles obtained with culture-dependent (isolation in culture) techniques indicated a low bacterial diversity. However, the same authors described higher bacterial diversity when using PCR-DGGE, a culture-independent method. These results from Lacava *et al*. (2007b) showed that PCR-DGGE is suitable for the analysis of bacterial diversity in GWSS heads. In the future, species such as *C. flaccumfaciens* and *Methylobacterium* spp., found as part of the bacterial community in GWSS, could be investigated as potential candidates for use in an SC or SC paratransgenic-based strategy to control the spread of *X. fastidiosa*.

Using methods perfected in previous studies (Lampe *et al*., 1999, 2000), Axd was genetically altered to contain a DsRed fluorescent marker gene in the chromosome (Bextine *et al*., 2004) to demonstrate the ability of DsRed Axd to colonize the cibarial region of the GWSS foregut for up to 5 weeks post-exposure. Axd was shown to occupy the same region in the foregut as the pathogen *X. fastidiosa* (Bextine *et al*., 2004). DsRed Axd was transmitted by GWSS and colonized various plants (Bextine *et al*., 2004, 2005). DsRed Axd could be introduced into grapevines by misting the leaves, by soil drenching or by direct injection into the stem of the grapevine. Interestingly, Axd appeared to be better adapted to citrus than to grapevine (Bextine *et al*., 2005). Indeed, the original samples of GWSS from southern California were obtained from citrus groves in the Agricultural Operations plots at the University of California, Riverside; therefore, it is likely that the endophytes in the GWSS originally came from citrus. Bextine *et al*. (2004) describe the successful delivery of Axd to, and the colonization of, the foregut of GWSS. These results suggest that a paratransgenic approach to manage, prevent, and/or control PD by SC may be possible.

A number of candidate antimicrobial peptides were screened against *X. fastidiosa* (Kuzina *et al*., 2006). In this study, the authors showed that antibiotics and antimicrobial peptides have some activity against *X. fastidiosa* and may have applications in protecting plants from developing PD. The potential use of these antimicrobial peptides in the protection of grapevines will depend on the development of a delivery system, such as SC (Kuzina *et al*., 2006). Additionally, Lampe *et al*. (1999, 2000) further screened single-chain antibodies from a phage antibody library for the ability to bind the coat protein of *X. fastidiosa*. These authors selected an antibody fragment, designated S1, that was specific to the strain of *X. fastidiosa* causing PD and did not recognize closely related *X. fastidiosa* strains.

Azizi *et al*. (2012) presented a simple robust approach for the generation of panels of recombinant single-chain antibodies against the surface-exposed elements of *X. fastidiosa* (PD) that may have potential use in diagnosis and/or disease transmission blocking studies. *In vitro* combinatorial antibody ribosome display libraries were assembled from immunoglobulin transcripts rescued from spleens of mice immunized with heat-killed *X. fastidiosa*. The libraries were used in a single round of selection against an outer membrane protein, MopB, resulting in the isolation of a panel of recombinant antibodies. The potential use of selected anti-MopB antibodies was demonstrated by the successful application of the 4XfMopB3 antibody in an enzyme-linked immunosorbent assay (ELISA), a western blot assay, and an immunofluorescence assay (IFA). These immortalized *in vitro* recombinant single-chain antibody libraries generated against heat-killed *X. fastidiosa* are a resource for the PD research community that may be readily accessed for the isolation of antibodies against a plethora of *X. fastidiosa* surface-exposed antigenic molecules.

Recently, Arora *et al*. (2015) reported a novel strategy for the delivery of genetically engineered bacteria in a paratransgenic system that targets the glassy-winged sharpshooter (*Homalodisca vitripennis*), an insect vector of grapes and citrus that transmits the phytopathogen *X. fastidiosa* (Dandekar *et al*., 2012; Rathe *et al*., 2014). Using simple and inexpensive materials for bioencapsulation (Weinbreck *et al*., 2010; Burgain *et al*., 2011) of the engineered symbiotic bacterium, *Pantoea agglomerans*, they demonstrated targeting of the sharpshooter *H. vitripennis* under controlled conditions with an alginate hydrogel that is tuned to release its bacterial payload during xylem flow into the foregut of the insect. By deploying a microencapsulation system that permits gated delivery of the bacterial payload to the arthropod, while greatly minimizing release in the environment, these authors concluded that robust field-applicable technologies for paratransgenic control of arthropod-borne diseases will be possible. According to these authors, this is the first example of the use of microencapsulation to deliver recombinant bacteria to an insect gut. They demonstrated that transgenic symbiotic bacteria can be delivered to the appropriate physiological niche within a disease-transmitting arthropod. Additionally, this platform may be expanded to deliver recombinant bacteria to other disease-transmitting arthropod vectors, thus facilitating field use of paratransgenic control.

## Strategy of symbiotic control for CVC

The key to symbiotic control is finding a candidate microbe with an existing association to the ecosystem that includes the problem or condition under investigation and that occupies the same niche as or has access to the target pathogen (Miller, 2011). Bacteria of the genus *Methylobacterium* are known to occupy the same niche as *X. fastidiosa* inside citrus plants (Araújo *et al*., 2002; Lacava, *et al*., 2004). During feeding, insects could acquire not only the pathogen but also endophytes from host plants. Gai *et al*. (2009) and Gai *et al*. (2011) reported the localization of the endophytic bacterium *M. mesophilicum* in the *C. roseus* model plant system and the transmission of this endophyte by *Bucephalogonia xanthophis*, a sharpshooter insect vector of *X. fastidiosa*.

*Methylobacterium mesophilicum*, originally isolated as an endophytic bacterium from citrus plants (Araújo *et al*., 2002), was genetically transformed to express GFP (Gai *et al*., 2009). The GFP-labeled strain of *M. mesophilicum* was inoculated into *C. roseus* (model plant) seedlings and was observed colonizing its xylem vessels. The transmission of *M. mesophilicum* by *B. xanthophis* was verified with insects feeding on fluids containing the GFP-labeled bacterium. Forty-five days after inoculation, the plants exhibited endophytic colonization by *M. mesophilicum*, confirming this bacterium as a nonpathogenic, xylem-associated endophyte (Gai *et al*., 2009). These data demonstrate that *M. mesophilicum* not only occupies the same niche as *X. fastidiosa* inside plants but also may be transmitted by *B. xanthophis*. The transmission, colonization and genetic manipulation of *M. mesophilicum* are prerequisites for it to potentially be used for paratransgenic SC to interrupt transmission of *X. fastidiosa* by insect vectors. We propose *M. mesophilicum* as a candidate for a paratransgenic SC strategy to reduce the spread of *X. fastidiosa*. It is known that *X. fastidiosa* produces a fastidian gum (da Silva *et al*., 2001), which may be responsible for the obstruction of xylem in affected plants (Lambais *et al*., 2000); therefore, the production of endoglucanase by genetically modified endophytic bacteria may transform the endophytes into symbiotic control agents for CVC (Ferreira Filho *et al*., 2012). Azevedo and Araújo (2003) have used the replicative vector pEGLA160 to produce genetically modified *Methylobacterium* expressing antibiotic resistance and endoglucanase genes. Furthermore, other strategies can be evaluated, such as the production of genetically modified *Methylobacterium* to secrete soluble anti-*Xylella* proteins in citrus. Lampe *et al*. (2006) suggested a similar strategy with the *Escherichia coli* α-hemolysin system for use in Axd to secrete soluble anti-*Xylella* protein effectors in grapevines and GWSS. Additionally, Lampe *et al*. (2007) suggested the evaluation of proteins secreted from the grapevine bacterial symbiont *P. agglomerans* for use as secretion partners of anti-*Xylella* protein effectors. One strategy that can be adopted as the next step for SC control of CVC is producing a genetically modified endophytic bacterium, such as *Methylobacterium*, to secrete anti-*Xylella* protein effectors.

Another strategy to control *X. fastidiosa* is to degrade the EPS (exopolysaccharides) formed by this plant pathogen that are directly related to biofilm formation. In *X. fastidiosa*, the fastidian gum may be directly linked to pathogenicity (da Silva *et al*., 2001) because it may be involved in the biofilm formation required for the attachment and survival of this bacterium in xylem vessels and the sucking pumps of the insect vectors. A lack of EPS would therefore prevent the plant symptoms caused by vessel occlusion (and/or embolism) and the spread of the disease as well (da Silva *et al*., 2001). Based on the premise of symbiotic control, Ferreira Filho *et al*. (2012) genetically modified the citrus endophytic bacterium *Methylobacterium extorquens*, strain AR1.6/2, and evaluated its capacity to colonize a model plant and its interaction with *X. fastidiosa*. The strain AR1.6/2 was genetically transformed to express heterologous GFP and endoglucanase A (EglA), generating the strains ARGFP and AREglA, respectively. Using fluorescence microscopy, it was shown that ARGFP was able to colonize the xylem vessels of the *C. roseus* seedlings. Using scanning electron microscopy, it was observed that AREglA and *X. fastidiosa* may co-inhabit the *C. roseus* vessels. *M. extorquens* was observed in the xylem with the phytopathogen *X. fastidiosa* and appeared to cause a decrease in biofilm formation. AREglA stimulated the production of the resistance protein catalase in the inoculated plants. These results demonstrate the successful transformation of AR1.6/2 to generate two different strains with a different gene and also indicate that AREglA and *X. fastidiosa* could interact inside the host plant, suggesting a possible strategy for the symbiotic control of CVC disease.

According to Ferreira Filho *et al*. (2012), the endophytic AR1.6/2 expressing the *EglA* or *gfp* genes showed most of the prerequisites listed by Durvasula *et al*. (2003) and Miller (2011) for a successful strategy of symbiotic control. For example, the AR1.6/2 strain that colonized citrus plants is amenable to isolation, culturing and transformation with foreign genes, and the heterologous expression of GFP and EglA by AR1.6/2 did not affect its growth and survival inside the host.

## Conclusions and Future Perspectives

Our strategy is similar to that developed by Bextine *et al*. (2004) for a paratransgenic strategy for SC of PD in grapevines. Bextine *et al*. (2004) suggested that the genus *Alcaligenes*, an endophytic bacterium that can colonize the GWSS vector of *X. fastidiosa*, would be a candidate for paratransgenic SC of PD in the USA. We believe that the endophytic bacterium *M. mesophilicum* from citrus plants is likewise a candidate for paratransgenic SC of CVC. Our results indicate that this endophyte colonizes the same niche as *X. fastidiosa* in citrus plants (Araújo *et al*., 2002; Lacava *et al*., 2004, 2006a; Andreote *et al*., 2006). *M. mesophilicum* is also transmitted by an insect vector of *X. fastidiosa* (Gai *et al*., 2009).

Bacteria chosen as gene delivery vehicles for paratransgenesis SC must not be pathogens themselves. *M. mesophilicum* is not a pathogen, and several requirements for a successful paratransgenesis SC strategy as described by Durvasula *et al*. (2003) and Miller (2011) have been demonstrated: a) *M. mesophilicum* is amenable to culture and genetic manipulation *in vitro*; b) facile methods for isolating and transforming the endophytic bacteria have been developed; c) transformation of the symbiotic/endophytic bacteria has resulted in mutants that were stable *in planta*. Future genetic manipulation of *M. mesophilicum* to achieve paratransgenic SC should not affect its symbiotic functions in the plant host and insect vector, and genetic manipulation of symbiotic bacteria should not render them virulent, either to the host plant or target.

*C. flaccumfaciens* is also a candidate for biological control of CVC. Interaction and antagonism between *C. flaccumfaciens* and *X. fastidiosa* was strongly indicated on the basis of the frequency of *C. flaccumfaciens* isolation from sweet orange (Araújo *et al*., 2002). In addition, *in vitro* interactions between *X. fastidiosa* and *C. flaccumfaciens* have been described, including the inhibition of *X. fastidiosa* growth by cell-free supernatants of nutrient medium in which *C. flaccumfaciens* had been grown (Lacava *et al*., 2004). Additionally, Lacava *et al*. (2007a) demonstrated that *C. flaccumfaciens* interacted with *X. fastidiosa* in *C. roseus* and reduced the severity of the disease symptoms induced by *X. fastidiosa* in this model plant to study the interaction of this plant pathogen and endophytic bacteria (Monteiro *et al*., 2001; Lacava *et al*., 2006a). The ability of *C. flaccumfaciens* to colonize plant tissues in the presence of *X. fastidiosa* and the reduction of disease symptoms caused by *X. fastidiosa* (Lacava *et al*., 2007a) are prerequisites for the use of this endophytic bacterium as a biocontrol agent. Because members of the genus *Curtobacterium* were consistently detected in the insect vectors of *X. fastidiosa* (Gai *et al*., 2011), they fulfill another requirement of candidates for biological control of *X. fastidiosa* (Lacava *et al*., 2007a), i.e., they can colonize both the insect vectors of *X. fastidiosa* and citrus plants. In the case of biocontrol of *X. fastidiosa* and CVC disease, it would be desirable if *C. flaccumfaciens* could be transmitted by budwood, but this has yet to be demonstrated. The reduction of disease symptoms caused by *X. fastidiosa* in the presence of *C. flaccumfaciens* may be attributable to direct killing of *X. fastidiosa* by *C. flaccumfaciens*. Consistent with this hypothesis, three bacteriocins showing activity against *X. fastidiosa* have been described in *C. flaccumfaciens* (Cursino, 2005, PhD thesis, University of São Paulo, Piracicaba, São Paulo, Brazil,).

**Figure 1:**
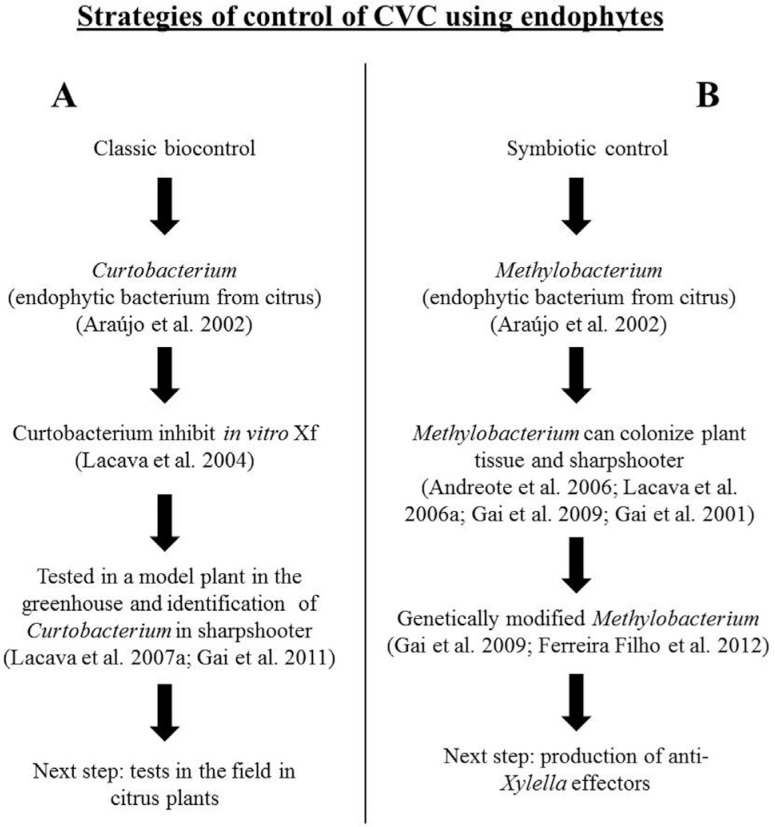
Hypotheses and strategies to control citrus variegated chlorosis (CVC) using endophytic bacteria from citrus plants. (A) We suggest the endophytic bacterium *Curtobacterium flaccumfaciens* as a classical biological control agent. *C. flaccumfaciens* has the ability to colonize plant tissues in the presence or absence of *Xylella fastidiosa* (Xf). This is a prerequisite for the use of this bacterium as a biocontrol agent. The data indicate that *C. flaccumfaciens* interacted with *X. fastidiosa* in *Catharanthus roseus* and reduced the severity of the disease symptoms induced by *X. fastidiosa* (Araújo *et al*., 2002; Lacava *et al*., 2004; Lacava *et al*., 2007a; Gai *et al*., 2011). (B) Additionally, the endophytic bacterium *Methylobacterium* has been suggested as a qualified candidate for a paratransgenic symbiotic control (SC) strategy because there have been reports on the transmission, colonization and genetic manipulation of *Methylobacterium*, which are prerequisites for the potential use of this bacteria to interrupt transmission of *X. fastidiosa*, the bacterial pathogen causing CVC, by insect vectors (Araújo *et al*., 2002; Andreote *et al*., 2006; Lacava *et al*., 2006a; Gai *et a*l. 2009, 2011; Ferreira Filho *et al*., 2012).

We propose two complementary strategies for control of CVC using endophytic bacteria from citrus plants. We suggest the endophytic bacterium *C. flaccumfaciens* as a classical biological control agent and the endophytic bacterium *M. mesophilicum* as a qualified candidate for a paratransgenic SC strategy. The details of these strategies are summarized in Figure 1.

**Figure 2:**
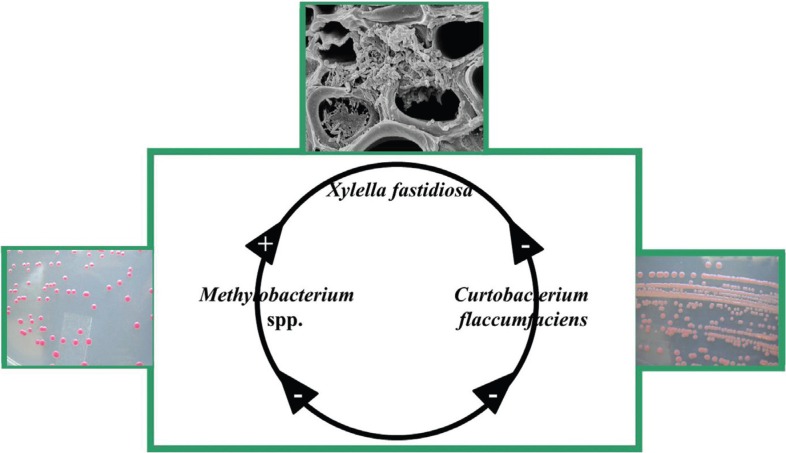
Balanced interactions among endophytic bacteria from *Citrus sinensis* and *Xylella fastidiosa*, the causal agent of citrus variegated chlorosis. Photos of endophytic strains of *Methylobacterium* and *Curtobacterium* grown in Petri dishes by the authors. Photo of scanning electron micrograph of the bacterium *X. fastidiosa* by E. W. Kitajima, ESALQ/USP/Brazil (http://aeg.lbi.ic.unicamp.br/xf/).

In addition to paratransgenic processes, the balance among endophytic microorganisms and *X. fastidiosa* is very important in the control of CVC. Approximately 15 years ago, a study conducted by the Microbial Genetics group in the Department of Genetics at Luiz de Queiroz College of Agriculture, University of São Paulo (ESALQ/USP), Brazil (Araujo *et al*., 2001, 2002) showed that in the same citrus plantations, *X. fastidiosa* is found in both symptomatic (showing disease symptoms) and asymptomatic plants (healthy plants). Although no genetic differences were found in these plants, distinctions were found in the composition of their endophytic bacteria. Bacteria of the genera *Curtobacterium* and *Methylobacterium* distinguished the endophytic communities of healthy and diseased plants, respectively. There may be many causes for this endophytic imbalance, including the use of agricultural chemical products, intensive cultures, distinct agricultural management and other abiotic and biotic factors (Laranjeira *et al*., 2005). It appears that the balance among endophytes, which has been maintained for thousands of years by co-evolution, can be altered, and some endophytic bacteria, such as *X. fastidiosa*, may become pathogenic due to this endophytic imbalance (Figure 2). Similar cases have been found; *Vitis vinifera* plants also display differences in disease severity and symptoms, and this was shown to be due to the presence of endophytic fungi, such as *Cochliobolus* sp., which inhibited *X. fastidiosa* by producing the antimicrobial radicinin. The absence of these fungi may result in the emergence of *X. fastidiosa* as a pathogen (Aldrich *et al*., 2015). Additionally, studies (Dourado *et al*., 2013, 2015; Lacava *et al*., 2004, 2006a) have shown that the development of CVC symptoms in citrus plants could be a result of an unbalanced endophytic population, including *Methylobacterium* spp. and *X. fastidiosa*. Therefore, the understanding of this interaction will allow the development of potential strategies to prevent CVC and other diseases caused not only by *X. fastidiosa* but also by other phytopathogens and physiological shifts, which may be due to the disequilibrium in the microbial community that is induced by agricultural management during crop production.
